# Targeting Cancer Stem Cells by Genetically Engineered Chimeric Antigen Receptor T Cells

**DOI:** 10.3389/fgene.2020.00312

**Published:** 2020-04-22

**Authors:** Rowa Y. Alhabbab

**Affiliations:** Division of Applied Medical Sciences, King Abdulaziz University, Jeddah, Saudi Arabia

**Keywords:** cancer stem cell, chimeric antigen receptor T cell, chimeric antigen receptor T cell production, chimeric antigen receptor generations, chimeric antigen receptor T cell signaling

## Abstract

The term cancer stem cell (CSC) starts 25 years ago with the evidence that CSC is a subpopulation of tumor cells that have renewal ability and can differentiate into several distinct linages. Therefore, CSCs play crucial role in the initiation and the maintenance of cancer. Moreover, it has been proposed throughout several studies that CSCs are behind the failure of the conventional chemo-/radiotherapy as well as cancer recurrence due to their ability to resist the therapy and their ability to re-regenerate. Thus, the need for targeted therapy to eliminate CSCs is crucial; for that reason, chimeric antigen receptor (CAR) T cells has currently been in use with high rate of success in leukemia and, to some degree, in patients with solid tumors. This review outlines the most common CSC populations and their common markers, in particular CD133, CD90, EpCAM, CD44, ALDH, and EGFR^VIII^, the interaction between CSCs and the immune system, CAR T cell genetic engineering and signaling, CAR T cells in targeting CSCs, and the barriers in using CAR T cells as immunotherapy to treat solid cancers.

## Introduction

Cancer stem cells (CSCs) were initially identified in acute myeloid leukemia (AML) and subsequently in several solid tumors such as breast, brain, gastric, and prostate tumors (Lapidot et al., 1994; Bonnet and Dick, 1997; Al-Hajj et al., 2003; Hemmati et al., 2003; Collins et al., 2005; Fukuda et al., 2009; Gupta et al., 2009). Although CSC represents a subpopulation from the total tumor cells, it is the engine that supports cancer growth (Batlle and Clevers, 2017). Therefore, CSCs are major obstacles in tumor treatment because even with the high effectiveness seen with the current chemo-/radiotherapy to remove most of the cancer cells, cancer patients usually suffer from relapse and cancer recurrence due to CSCs resistance, renewal, and differentiation ability initiating new tumor in treated patients (Reya et al., 2001; Hong et al., 2015; Kaiser, 2015; Kaur G. et al., 2018). Thus, therapeutic approaches to eliminate CSCs are a necessity to overcome relapse and cancer recurrence in those patients.

Advances in immunotherapy and the development of chimeric antigen receptor (CAR) T cells have provided a solid and successful approach to target any protein expressed by cancer cells. CAR T cells’ cytolytic capacity is independent of the major histocompatibility complex (MHC), and they are genetically engineered to express a target-specific antigen receptor (June and Sadelain, 2018). Clinically, a large number of patients with large B cell lymphoma (LBCL) and B cell acute lymphoblastic leukemia (B-ALL) have shown total remission when treated with a single CAR T cell infusion (Maude et al., 2014b, 2018; Lee et al., 2015; Turtle et al., 2016a, b; Gardner et al., 2017; Neelapu et al., 2017; Fry et al., 2018; Park J.H. et al., 2018; Schuster et al., 2019). However, targeting solid tumors with CAR T cells was not associated with the same robust outcomes, but hope of success originates from some associated efficiency seen during early signs of clinical trials (Majzner and Mackall, 2019). Therefore, to use CAR T cells as a therapy to target CSCs, many efforts have been made to identify several markers to distinguish CSCs from other cancer cells (Codd et al., 2018). In the present review, CSC populations as well as their most common markers, the interaction between CSCs and the immune system, CAR T cells bioengineering and signaling pathways, clinical applications in targeting CSCs using immunotherapeutic approaches, in particular CAR T cells, and the barriers in using CAR T cells are discussed.

## CSC Populations and Common Markers

Tumor heterogeneity and development have been described in two models, the clonal evolution and CSC models (Marjanovic et al., 2013). The clonal evolution model proposes that stochastic events enable the selection and the advantageous growth of colonies that arose from the continuous acquisition of accumulated mutations. On the other hand, the CSC model suggests that particular tumor cells, which have the capacity to activate the expression of stem cell genes, are capable of driving tumor progression. These cells are thought to divide through asymmetric division, leading to the semipreservation of the parental cell genotype and the generation of a daughter cell that may pose novel mutations and not necessarily express stem cell genes. This controlled aspect of division is thought to enable the increase in heterogeneity in a hierarchical manner. CSCs may be rarer and less heterogeneous in early developed low-grade tumors (Alamir et al., 2018). In contrast, high-grade progressive tumors often have a highly varied heterogeneous population of CSCs, perhaps due to a weakened control on asymmetric cell division as more mutations are accumulated (Khan et al., 2018). Importantly, CSCs are tightly associated with the ability to initiate metastatic tumors and are inclined to be drug resistant (Aydemir Coban and Sahin, 2018). The CSCs model is gaining scientific popularity, as the clonal model is not always applicable to the formation of human cancers and does not sufficiently clarify the differences in the level of cancer heterogeneity between grades. Therefore, some have suggested dropping this model (Afify and Seno, 2019). However, for more details about clonal evolution, readers are referred to Marjanovic et al. (2013) and Afify and Seno (2019).

CSCs share many functional features with healthy stem cells including the ability to regenerate and proliferate extensively (Badrinath and Yoo, 2019). Although all types of CSCs identified until now have shared these properties as well as their resistance to the current therapy, each population identified in different tumor types such as breast, colon, brain, and leukemia has a unique marker and driver pathway (Desai et al., 2019). CSCs were identified 25 years ago in AML though transplanting the initiating AML cells into immunodeficient mice (SCID). These cells resided and proliferated in the bone marrow in response to cytokines treatment and generated leukemic cells similar in morphology to their counterpart in the original patients. Moreover, they found that these AML-initiating cells were CD34^+^CD38^–^ (Lapidot et al., 1994). Subsequently, several surface markers have been identified to distinguish leukemia stem cells (LSCs) including CD123, TIM3, CD47, CD96, CLL-1, and IL1RAP (Blair and Sutherland, 2000; Hosen et al., 2007; [Bibr B333][Bibr B142]; Kikushige et al., 2010; Askmyr et al., 2013; Bruserud et al., 2017). The identification of these LSC surface markers has led to the generation of several promising therapeutic approaches targeting LSC of several hematopoietic malignancies, in particular those expressing CD123 (Busfield et al., 2014; Frankel et al., 2014; He et al., 2015; Chichili et al., 2015; Al-Hussaini et al., 2016; Ruella et al., 2016).

The first CSCs identified in solid tumor were of the breast tumor. These CSCs were characterized by the expression of CD44 and low levels of CD24 (Al-Hajj et al., 2003). Although several successful approaches have been reported in targeting brain CSCs (BCSCs), none of these therapies has been approved for targeting BCSCs (Desai et al., 2019). Ignatova et al. (2002) were the first to describe the brain CSCs. Since then, several characteristic markers for brain CSC have been documented including CD133 (Hemmati et al., 2003), CD49 (Lathia et al., 2010), L1CAM (Bao et al., 2012), and CD36 (Hale et al., 2014). Although the expression of these markers are different between patients and not sufficient on their own to designate brain CSC population, these markers are broadly used to identify adult brain CSCs (Desai et al., 2019). Moreover, CD133 and CD49 are expressed on both adult and pediatric brain CSCs regardless of the fact that both diseases are considered different. Therefore, targeting brain CSCs expressing CD133 in adults would provide a different outcome upon using the same approach with pediatrics (Desai et al., 2019). Colon CSCs share a phenotypic marker with the brain CSCs in which both were identified to express CD133 (Ricci-Vitiani et al., 2007), however, colon CSCs have been reported to express CD44 (Cheng et al., 2006), CD26 (Pang et al., 2010), as well as LGR5 (Schepers et al., 2012). Although preclinical trial targeting CD133-expressing cell has been a success (Ning et al., 2016), using combining therapies targeting both LGR5^+^ colon CSCs and the differentiated tumor cells could show more success and prevent patient relapse (Shimokawa et al., 2017). CSC populations of other cancer types have also been described expressing different markers, and targeting these cells is considered as a promising therapy to treat the disease. The general features of the most commonly known markers to isolate solid cancer CSCs are discussed below.

### CD133

CD133 is one of the most commonly used markers to identify CSCs of different tumors. CD133 is a product of a single-copy gene on chromosome 4 (4p15.33) in humans. The human gene consists of at least 37 exons spanning ∼160 kb. The transcript size is ∼4.4 kb. The transmembrane glycoprotein consists of 865 amino acids (aa) with a total molecular weight of 120 kDa. CD133 consists of five transmembrane glycoproteins. Despite that little is known about CD133 function, it has been reported to bind to cholesterol and found to be in the membrane protrusions (Visvader and Lindeman, 2008; Codd et al., 2018). Although CD133 has been accepted as a marker for CSCs, however, CD133 expression varies depending on the type of cancer, and it could be expressed on several noninitiating cancer cells as well as several healthy tissues and healthy stem cells (Shmelkov et al., 2008; Zhou et al., 2011). Therefore, CD133 cannot be used alone as a specific marker for CSCs. Moreover, using CD133 to detect CSCs has led to some inconsistent outcomes that might be due to their expression array and the detecting antibodies used (Hermansen et al., 2011). The antibodies to detect CD133 is usually mouse monoclonal antibodies against two different glycosylated epitopes, AC133 and AC141; therefore, the variation in the level of their glycosylation among the tissues could lead to false negative results (Codd et al., 2018).

### CD90 and EpCAM

CD90 is a plasma membrane glycophosphatidylinositol anchor protein and is expressed in several tissues including skin and tissues of both the nervous as well as the olfactory systems (Sauzay et al., 2019). Recently, it has also been reported that CD90 is a marker expressed on the stem cells of the epidermis, liver, hematopoietic, and mesenchyme (Kumar et al., 2016). Moreover, several ligands for CD90 have been identified such as CD97, αv/β3, syndecan-4, CD90, and αx/β2 (Wandel et al., 2012; Kong et al., 2013; Leyton and Hagood, 2014). CD90 mainly function as an adhesion molecule, however, it is also involved in many other physiological functions including nerve regeneration and growth, migration as well as adhesion of leukocytes, apoptosis and activation of T cells, migration, and proliferation of the fibroblast (Rege and Hagood, 2006; Barker and Hagood, 2009; Bradley et al., 2009; Leyton and Hagood, 2014). Nowadays, CD90 is considered as a marker for CSCs in gastric and esophageal squamous cell carcinomas and hepatocellular carcinoma (HCC) due to the ability of tumor-isolated CD90^+^ cells to generate cancer even upon the adoptive transfer of a very small number of these cells into immunodeficient mice compared to tumor-isolated CD90^–^ cells (Yang et al., 2008; Jiang et al., 2012; Tang et al., 2013). Moreover, CD90^+^ cells isolated from gliomas, lung, esophageal squamous cell carcinomas, and gastric cancers were able to regenerate and grow as a spheroid’s *in vitro* serum free media (Kang and Kang, 2007; He et al., 2012; Jiang et al., 2012; Tang et al., 2013; Wang P. et al., 2013).

EpCAM is a transmembrane glycoprotein and is involved in cell adhesion as well as cells proliferation, differentiation, migration, signaling, and regeneration (Keller et al., 2019). Several studies have been using EpCAM plus CD44 as a marker for CSCs including CSC found in the liver, breast, prostate, colon, and pancreatic cancers (Yamashita et al., 2007; Gires et al., 2009).

### CD44

CD44 is another common marker to identify CSCs in various cancer types, similar to CD133 and EpCAM. It is transmembrane glycoprotein, however, it has several functions such as a receptor for hyaluronic acid, as well as the ability to be involve in the adhesion, migration, proliferation. and survival of cells (Codd et al., 2018). Unfortunately, as with the abovementioned markers, CD44 is also expressed on healthy cells, making it difficult to be used to specifically differentiate CSCs. However, the ability of CD44 encoding gene to express multiple isoforms including CD44v, CD44s, and other variants gave the opportunity to identify that CD44v is highly expressed on tumor-capable cells compared to CD44s, while other variants have been identified to be associated with the progression of several cancer types (Mashita et al., 2014; Todaro et al., 2014; Thapa and Wilson, 2016). Furthermore, in head and neck cancer, it was found that tumor cells expressing high levels of CD44 are less immunogenic than CD44^lo^ cells. The latter was associated to the PD-L1 high expression by CD44^hi^ cells (Lee et al., 2016). Targeting CD44 binding domain by IgG1 antibodies during clinical trials showed high level of safety but modest effect in patients. This might be due to the crucial role that CD44 plays in T cells, in particular T helper (Th) 1 cells, in the proliferation, survival, memory function, and proinflammatory cytokines production (Baaten et al., 2010; Schumann et al., 2015; Menke-van der Houven van Oordt et al., 2016).

### ALDH

Aldehyde dehydrogenase (ALDH) is a superfamily of 19 human isozymes and highly expressed in healthy as well as cancer cells with stem-like characteristics, however, ALDH expression is not limited to stem cells but also can be expressed by mature cells (Fillmore and Kuperwasser, 2008; Xu et al., 2015; Vassalli, 2019). ALDH is an enzyme that has the ability to oxide varied range of aldehydes, endogenous and exogenous, to their carboxylic acids to provide protection against oxidative stress. Moreover, ALDH have the ability to regulate cellular homeostasis through its role in the biosynthesis of the responsible molecules including retinoic acid (Marchitti et al., 2008; Jackson et al., 2011; Vassalli, 2019). ALDH roles have made it an attractive molecule in studying CSCs; therefore, many reports have identified ALDH as a specific marker for CSCs in several cancers. Moreover, healthy stem cells and CSCs can be differentiated by measuring the catalytic activity of ALDH that can also be used to monitor the prognosis of certain cancer patients (Ginestier et al., 2007; Deng et al., 2010; van den Hoogen et al., 2010; Marcato et al., 2011; Silva et al., 2011; Singh et al., 2015). With regard to ALDH association with stem cells, most of the focus has been placed on ALDH members that play role in the biosynthesis of retinoic acid via their cytosolic enzyme activity such as ALDH1 (Vassalli, 2019). ALDH1A1 is highly expressed by malignant CSCs in several cancers (Xu et al., 2015). Moreover, CSC uses ALDH to survive chemotherapy by blocking signal transducer and activator of transcription 3 (STAT3)–nuclear factor kappa B (NF-κB) signaling, a pathway that can diminish the accumulation of ALDH1A1 and sensitize tumor cells to chemotherapy (Canino et al., 2015; Zhao, 2016).

### EGFR^VIII^

Epidermal growth factor receptor (EGFR) is a transmembrane glycoprotein with a molecular mass ranging from 170 to 185 kDa (Weingaertner et al., 2013). Thirteen legends have been identified for EGFR activation such as epidermal growth factor (EGF); generally, activation via EGFR initiates several signaling pathways including Ras/Raf/mitogen-activated protein kinase (MAPK), phosphatidylinositol-4,5-biphosphate 3-kinase (PI3K)/AKT, Janus kinase (JAK)/STAT, or phospholipase C (PLC)/protein kinase C (PKC) (Harris et al., 2003). Therefore, EGFR activation is involved in several cellular processes such as cell survival, proliferation, differentiation, apoptosis, and metabolism (Mendoza et al., 2011; van de Water et al., 2012; Jones and Rappoport, 2014; Treda et al., 2016). Several tumor-associated mutations of the EGFR gene have been identified. These include EGFR^VI^ for the deletion of the N-terminal part, EGFR^VII^ for the deletion of exons 14 and 15, EGFR^VIII^ for the deletion of exons 2–7, EGFR^VIV^ for the deletion of exons 25–27, and EGFR^VV^ for the deletion of exons 5–28 (Wong et al., 1992; Cho et al., 2011; Guillaudeau et al., 2012; Francis et al., 2014). EGFR mutations are usually accompanied with prolonged signaling that is associated with metastasis, angiogenesis, apoptosis inhibition, and enhanced proliferation of the tumor cells (Nagane et al., 1996; Sangar et al., 2014). The EGFR amplification is associated with most of the glioblastoma (GBM) cases, with EGFR^VIII^ being the most detected variant (Yamazaki et al., 1990; Wikstrand et al., 1995; Voldborg et al., 1997; Okamoto et al., 2003). In fact, EGFR gene is amplified in ∼50% of GBM patients, with 50–60% of the patients expressing EGFR^VIII^. Moreover, EGFR^VIII^ is rarely expressed in healthy tissues, making this exclusive tumor-mutated receptor an attractive therapeutic molecule (Wong et al., 1992; Moscatello et al., 1995; Del Vecchio and Wong, 2010; Snuderl et al., 2011; Del Vecchio et al., 2013).

Altogether, CSC markers have been shown to be useful for CSC enrichment. However, their utilization is limited due to the variability seen in their expression, which is perhaps caused by variation in the tumor microenvironment (TME). For instance, CD133 accuracy as a phenotypic marker for CSC is still controversial, in which several studies found that CD133^+^ tissues are capable of regenerating tumor population with heterogenic properties *in vitro* and *in vivo*, whereas others reported that GBM cells expressing CD133 and CD133^–^ cells have equal potential to generate tumor when transferred into nude mice (Singh et al., 2003, 2004; Beier et al., 2007). Moreover, it has been reported that some differentiated cancer cells have the ability to acquire stem-like characteristics displaying a great degree of phenotypic plasticity (Brooks et al., 2015). In breast cancer, two CSC subpopulations identified by ALDH1^+^ and CD44^+^ were found to have the potential to interconvert between themselves and with ALDH1^–^ as well as CD44^–^ nonCSCs (Liu S.L. et al., 2014). Therefore, it is crucial to understand the molecular foundations that regulate the expression of CSC markers and clarify their roles in maintaining CSC. Nevertheless, it is important to continue to uncover the nature of CSC markers, since their expression has been shown to correlate with patient survival in various types of solid cancers. Notably, CSC plasticity and heterogeneity are one of the challenging barriers that effect the patient’s response to CAR T-cell therapy.

## Immunity and CSCs

The components of the immune system play a complicated role in CSCs development. Macrophages are one of the most important cells of the innate immune system and can be polarized either into M1 or M2 macrophages (Ley, 2017). M1’s main function is to defend the host by killing pathogens, virally infected cells, as well as cancer cells, while M2 clears the eliminated invaders by M1 and repairs the damage associated with the process of pathogen killing (Mills, 2012; Ley, 2017). M2 macrophages have also been reported to have mutual supportive relation with CSC development and growth. For instance, Jinushi et al. (2011) have reported that milk-fat globule EGF-8 (MFG-E8) producing M2 macrophages promote CSC resistance to anticancer drugs and tumorigenicity by activating their Sonic Hedgehog signals and Stat3 pathway. In addition to M2 macrophages’ production of MFG-E8, M2 macrophages were also reported by Jinushi et al. (2011), to produce interleukin-6 (IL-6) that supports the same role as MFG-E8 in triggering CSCs’ tumorigenicity and resistance to therapy. Moreover, it has been proposed that CSCs can enter latency stage and escape natural killer (NK) cells killing mechanism through downregulating the ligand that activate NK cells by expressing DKK1, a WNT pathway inhibitor (Malladi et al., 2016). It has also been reported that neutrophil extracellular trap released from activated neutrophils due to sustained lung inflammation can waken dormant tumor cells and initiate metastasis as well as cancer growth (Albrengues et al., 2018). These data support the notion of the importance of the interaction between CSCs and the immune system, however, since the reports are limited, more evidence are required to clarify and draw the whole picture of their interactions.

Generally, CSCs are immunosuppressive and can escape the immune system through several mechanisms to maintain their survival and establish resistant and heterogenic tumor (Prager et al., 2019). For instance, some CSCs escape the cytotoxic T cell killing process by downregulating their MHC class I (Di Tomaso et al., 2010; Schatton et al., 2010) or by decreasing their antigen processing capacity by reducing their low molecular weight protein and transporter associated with antigen processing (Di Tomaso et al., 2010). Furthermore, it has been reported that CSCs can partially mimic the expression of both their MHC class I and their inhibitory costimulatory molecules, such as PD-L1, with absences in the expression of their activating costimulatory molecules including CD80, CD86, and CD40. Upon contact with effector T cells, this improper stimulation induces effector T cells’ anergy (Silver et al., 2016). This was supported by Parsa et al. (2007), in which they found that PD-L1 expressing tumor cells inhibited the activation and cytokine production by effector T cells via their direct interaction. An additional interesting mechanism was reported by Wei et al. (2010), in which they found that CSCs of GBM can induce naive as well as activated T cell apoptosis through galectin-3 secretion, allowing CSC expansion and depleting the intratumor effector cells of the immune system. It has also been reported that CSCs produce several anti-inflammatory cytokines including transforming growth factor beta (TGF-β) and IL-4 (Nappo et al., 2017; Prager et al., 2019). TGF-β is well known as an inducer for both Tregs via FoxP3-independent and FoxP3-dependent pathways as well as pro-oncogenic M2 macrophages, to prevent effector T cell proliferation and to inactivate NK cells (Fantini et al., 2004; Thomas and Massague, 2005; Oh et al., 2017). M2 macrophages are induced by cancer cells and produce high levels of cytokines, express several enzymes including arginase 1 as well as protease and growth factors, all together promoting tumor growth and immunosuppression (Solinas et al., 2010; Weng et al., 2019). CSCs promote these cells’ differentiation and recruitment from blood monocytes by producing periostin (Zhou et al., 2015) or direct interaction via CD90-CD11b and EphA4-Ephrin (Lu et al., 2014). Moreover, it has been reported that CSCs express inhibitory receptors such as CLTA-4 and PDL-1 on their surface to induce immunosuppressive cells. Although blocking those molecules has shown great success in clinical trials (Pardoll, 2012; Li S. et al., 2018), PDL-1 expression by CSCs is controversial, in which some studies reported PDL-1 expression on CSCs while others found it undetectable (Maccalli et al., 2014). Therefore, more studies are required to investigate other CSCs’ immune evasion mechanisms to minimize tumor recurrence and metastasis. Table 1 summarizes the various CSC identified mechanisms to modulate the immune system.

**TABLE 1:** The various published mechanisms used by cancer stem cells (CSCs) to modulate the immune system responses.

Mechanisms by CSC to modulate the immune system responses		

1. Altering surface molecules expression		

Surface molecules	Modulation	References
a. MHC I, MHC II, and NKG2D ligand molecules	Decreasing MHC I and II without expressing NKG2D ligand molecules lower CSC immunogenicity and increase their immunosuppressive activities.	Di Tomaso et al., 2010
b. B7-H1 (PD-L1) and galectin-3	Increased expression of PD-L1 and secretion of galectin-3 by CSCs induces Tregs and inhibits the proliferation of effector T cells.	Wei et al., 2010
c. TLR-4	Reducing TLR-4 expression by CSCs elevates retinoblastoma-binding protein 5 that activates CSCs self-renewal ability.	Alvarado et al., 2017
d. MICA and MICB (ligands for stimulatory NK cell receptor: NKG2D).	Reducing MICA and MICB expression promote CSCs resistance to NK cytotoxic killing.	Wang et al., 2014
e. CD47	Overexpression of CD47 promotes CSC escape from bone marrow-derived macrophages phagocytosis.	Zhang et al., 2015
f. PD-L1	High expression of PD-L1by CSC induce T cell anergy and Tregs differentiation.	Hsu et al., 2018
g. CD133 and CXCR4	CD133 and CXCR4 expression by CSCs increase their tumorigenicity, metastasis and resistance to therapy.	Hermann et al., 2007

**2. Secretion of anti-inflammatory molecules**		

**Secreted molecules**	**Modulation**	**References**

a. Macrophages inhibitory cytokine 1 (MIC-1)	Production of MIC-1 by CSCs inhibit phagocytosis by macrophages and suppress T cell proliferation.	Wu et al., 2010
b. Macrophage migration inhibitory factor (MIF)	MIF secretion by CSC induces arginase 1 production from MDSC (myeloid-derived suppressor cell) that in turn inhibit antitumor T cell responses.	Otvos et al., 2016
c. IL-4	IL-4 production by CSCs enhances cancer growth, resistance to therapy and mediate effector T cells suppression.	Todaro et al., 2007; Volonte et al., 2014
d. TGF- β	TGF-β secretion by CSCs induces Tregs and M2 macrophages and prevent effector T cell proliferation and inactivate NK cells.	Fantini et al., 2004; Oh et al., 2017; Thomas and Massague, 2005

The immune system can eliminate CSCs either through antigen nonspecific mechanisms or through antigen-specific targeting-dependent approaches. NK cells are known for their ability to target and eliminate normal mesenchymal stem cells as well as various CSCs (Jewett et al., 2013; Ames et al., 2015a). This was seen in several studies targeting different types of CSCs, including GB, pancreatic, melanoma, oral, and lung CSCs; these studies documented that the main immune effector cells capable of targeting all these types of CSCs are the NK cells (Bui et al., 2015; Kozlowska et al., 2016, 2017). Moreover, NK cells are well known for their crucial role in killing cancer cells nonspecifically via recognizing the downregulation in the level of MHC class I (inhibitory signals) with the upregulation in the expression of the legends for NK-cell-activating receptors (activating signals) on the surface of the cancer cells. This equilibrium between NK cells activating and inhibitory signals is required for NK cell activation and effective antitumor killing function. Cancer cells are highly susceptible to NK cells killing, in particular, CSCs because they express lower levels of MHC class I than the rest of the tumor cells (Codd et al., 2018). However, some CSCs that are associated with certain cancer types can resist NK cell killing because they do not express NK-cell-activating legends (Wu et al., 2007; Wang et al., 2014). On the other hand, some CSCs express low levels of MHC class I as well as high levels of NK-cell-activating markers and therefore are more susceptible to killing by NK cells (Castriconi et al., 2009; Tseng et al., 2010; Tallerico et al., 2013).

CSCs can be identified from tumor-differentiated cells by MHC class I negative or decreased levels, CD54, PD-L1, as well as an increase in CD44 expression (Bui et al., 2015; Kozlowska et al., 2016). Jewett et al., have identified a maturational stage of NK cells in which the cells’ CD16 expression levels are downregulated. NK cells at this stage of development were also characterized by their reduced cytotoxic ability upon interaction with CSCs, while interferon gamma (IFN-γ) and tumor necrosis factor alpha (TNF-α) production is maintained, a functional state identified as “split anergy” (Jewett et al., 1997; Tseng et al., 2015a; Jewett et al., 2008). This functional state is reported to be essential for the tumor differentiation and potential NK cell inactivation (Bonavida et al., 1993; Jewett and Bonavida, 1996; Tseng et al., 2015a). Supernatants obtained from split anergy NK cells were reported to mediate CSC differentiation mainly via IFN-γ and TNF-α, which in turn were documented to reduce the degree of tumor growth and induce tumor cell resistance to NK cell killing (Tseng et al., 2014, 2015a,b; Bui et al., 2015; Kaur K. et al., 2018). This was found to be associated with an increase in MHC class I, PD-L1, and CD54 expression and a reduction in CD44 levels on tumor cells. This was confirmed through adding anti-IFN-γ and anti-TNF-α antibodies to stimulated NK cells prior to their utilization in tumor differentiation; the antibodies inhibited the upregulation of these markers on the cancer cells (Tseng et al., 2014, 2015a,b). In addition, Ames and colleagues have reported that CSCs from various cell lines, as well as those isolated from primary tumor specimens based on the expression of several CSC markers including CD24, CD44, CD133, and ALDH, are eliminated preferentially by activated NK cells. This was dependent on the expression of several NK cell activation markers on CSCs including MICA/B, Fas, and Death receptor 5. Moreover, adoptive transfer studies have shown that the adoptive transfer of stimulated NK cells into orthotopic human pancreatic cancer tumor-bearing mice significantly reduced intratumoral CSCs as well as tumor burden (Ames et al., 2015a). The same group have also published that *ex vivo* stimulated NK cells are capable of targeting solid cancers CSCs *in vitro* postCSCs radiation, which was found to increase the number of CSCs expressing stress ligands such as MICA/B and Fas. Upon adoptive transfer along with radiotherapy, locally radiated tumor-bearing mice survival was prolonged (Ames et al., 2015b). Although CSCs are highly susceptible to NK cell killing, the report of Castriconi et al. (2009), shows that NK cells isolated from GBM patients are incapable of killing CSCs, despite that cytokines activated NK cells isolated from healthy donors were able to eliminate CSCs. These data points at the importance of the TME in NK cell function in killing CSCs, as well as their possible role in modulating CSC phenotype to evade NK cell’s killing mechanisms.

TME plays a curtail role in NK-cell-mediated cytotoxicity and can prevent NK cell function via two major approaches: suppression of NK cells and evasion via immunoediting of the tumor cells. At the tumor site, the TME favors type 2 over type 1 responses that may suppress the infiltrated NK cells upon their interactions with tumor (Vitale et al., 2014). Tumor-associated cells residing at the tumor site, including immature dendritic cells (DCs), Tregs, tumor-associated macrophages, and myeloid-derived suppressor cells, produce various molecules such as TGF-β, IL-4, IL-10, prostaglandin E2, and idoleamine 2,3-dioxygenase (Stojanovic et al., 2013; Konjevic et al., 2019). These molecules enable the tumor to downregulate NK-cell-activating receptors including NKp30, NKp44, or NKG2D, as well as tumor necrosis factor-related apoptosis-inducing ligand (Baginska et al., 2013; Vitale et al., 2014; Zenarruzabeitia et al., 2017; Park A. et al., 2018; Nayyar et al., 2019; Konjevic et al., 2019). For instance, TGF-β can inhibit the expression of NK cell receptors including NKp30 and NKG2D, which is essential for tumor recognition and elimination by NK cells and for their productive interaction with DCs (Castriconi et al., 2003). Similarly, NK cells’ potential to eliminate tumor cells and functional interaction with DCs can be reduced by IL-4 produced and released into the TME (Marcenaro et al., 2005). Besides molecule production by tumor residence cells, immune cells at the tumor site can modulate NK cell function by competing for IL-2 or inhibiting NK cell IL-2-mediated activation via cell-to-cell contact (Sitrin et al., 2013; Sprinzl et al., 2013). TME is often associated with hypoxia, which has been reported to significantly suppress both the expression and function of NK cells’ major activating receptors (Balsamo et al., 2013). As mentioned earlier, tumor cells can evade NK cells via immunoediting, which can occur due to chronic exposure of tumor cells to NK cells. For example, tumor-resistant melanoma cells cocultured with NK cells displayed an increased level of MHC class I (Balsamo et al., 2012). Collectively, these mechanisms could disturb the equilibrium between NK cell activation and inhibitory signals. Several other TME factors are reported to modulate NK cell cytotoxic function including the TME influence on NK cell metabolism. However, NK cells are not the focus of this review; therefore, for full comprehensive discussion, readers are referred to Terren et al. (2019) and Chambers et al. (2018).

T-cell receptor (TCR) divides the T cells into two populations: αβ TCR and γδ TCR T cells. Unlike αβ T cells that are MHC-dependent, γδ T cell activation is direct and independent of MHC molecules (Sebestyen et al., 2019). The protective role of γδ T cells in cancer was first reported in a mouse model of cutaneous squamous cell carcinoma, in which the adoptive transfer of γδ T cells into mice deficient of γδ T cells prevent the cancer development (Girardi et al., 2001). Subsequently, several studies reported the key protective role that γδ T cells play in preventing cancer. γδ T cell protection against cancer is mainly reported to be through the production of proinflammatory cytokines such as IFN-γ, TNF-α, and IL-17 as well as through their cytotoxic ability (Ma et al., 2011; Sebestyen et al., 2019). However, clinical trials stimulating γδ T cells or even transferring γδ T cells with or without activating stimuli into cancer patients show very low efficiency and very limited success (Wilhelm et al., 2003; Dieli et al., 2007; Bennouna et al., 2010; Meraviglia et al., 2010; Nakajima et al., 2010; Lang et al., 2011; Kobayashi et al., 2011; Kunzmann et al., 2012; Bregeon et al., 2012; Wada et al., 2014; Pressey et al., 2016; Aoki et al., 2017). This might be due to the lack of knowledge regarding the specificity and diversity of these cells. γδ T cells are characterized by their ability to recognize early metabolic changes including stress-induced self-antigens that differentiate healthy cells from transforming one. Therefore, identifying the proper activating process of γδ T cells as well as their receptors would lead to successful identification of tumor cells with very low mutational changes at early stages, unlike any other immunotherapeutic approaches (Sebestyen et al., 2019). As mentioned earlier, the adoptive transfer of γδ T cells into cancer patients was not that successful but was associated with high level of safety; therefore, γδ T cells are currently suggested to be used as CAR carriers (Fisher and Anderson, 2018; Liu et al., 2019). Similar to antitumor CAR NK cells that have been reported to be associated with less harmful side effects, such as cytokine release syndrome (CRS), γδ T cells are postulated to be associated with the same level of safety (Li Y. et al., 2018). Nevertheless, γδ T cells and NK cells can eventually be educated due to their tight control by several receptors such as natural cytotoxicity and killer-cell immunoglobulin-like receptors (Orr and Lanier, 2010). Additional probable issue with using γδ T cells as a CAR carrier cells is the possible long survival of these cells, as has been documented for the NK cells; moreover, the metabolic changes that γδ T cells recognize can occur in normal cells postexposure to stressful conditions (de Witte et al., 2018). Furthermore, using γδ T cells as a CAR carrier will not clear up the issue of identifying target independent of the changes load that γδ T cells recognize in transforming cells (Hartmann et al., 2017; Sebestyen et al., 2019).

CD8 T cells represent the major tumor killer cells of the adoptive immune system. Generally, cancer cells including CSCs express MHC class I but not MHC class II, and CD8 T cells recognize cancer antigens in a specific manner depending on the proper presentation of antigens on MHC class I as well as on the level of MHC class I (Codd et al., 2018). However, CSC targeting by CD8 T cells has been reported to be either resistant or susceptible to T cell killing depending on the type of cancer and origin and culture conditions of the cells (Codd et al., 2018). Several antigens have been documented to be specifically expressed on MHC class I of the CSCs such as cancer/testis (CT) antigens. CT antigens are expressed exclusively on germ cells but can reappear in some cancer cells (Codd et al., 2018). One example of CT antigens that have been found to be solely expressed on CSCs is the brother of the regulator of the imprinted site (BORIS), which is found to be expressed on CSCs of cervical as well as lung cancers, and can be targeted successfully by specific CD8 T cells (Asano et al., 2016; Horibe et al., 2017). CT antigens are classified as one of the tumor-associated antigen (TAA) family, however, for a full comprehensive review on TAA as well as CT antigens, the reader can refer to this reference Hirohashi et al. (2016).

## Csc-Specific Targeting by Car T Cells in Clinical Applications

Several immunotherapeutic approaches to treat cancers have been developed including monoclonal antibodies, adoptive T cell therapy, immune checkpoint inhibitors, oncolytic virus therapy, and cancer vaccines. All of these therapies are still under extensive investigations and are associated with shared advantages as well as disadvantages. Immunotherapeutic medicine is characterized and differs from the traditional cancer therapy by being highly selective to tumor cells and is not associated with unpleasant side effects. Although immunotherapies are not free from adverse side effects, as these therapies are developing and evolving, the side effects become more controllable. Moreover, immunotherapies can stimulate the immune system against cancer for a long period and, therefore, might provide long-term remission and reduce tumor recurrence. However, the long-term influence and efficiencies are still unclear. As the immune system has the ability to eliminate almost all types of cancer cells, designing immunotherapy that allow immunity to perform such a function will be a very beneficial challenge to overcome. As with many treatments, immunotherapies are associated with some disadvantages, and one of the major obstacles is the high cost and the intensive labor required to produce the treatment. Immune checkpoint inhibitors are considered the most attractive treatment among all of the available immunotherapies due to the long-term benefits seen in melanoma, Hodgkin’s lymphoma, and Merkel cell carcinoma patients (Schmidt, 2017). Nevertheless, similar to CAR T cell treatment and other clinically used immunotherapies, immune checkpoint inhibitors are not beneficial to all patients and the benefited patients can suffer from acquired resistance. Generally, acquired resistance includes loss of target antigens, particularly seen with T cell adoptive therapies, upregulation of the expression of immune checkpoint legends such as PD-L1 on target cells, and accumulation in Tregs at TME (Sharma et al., 2017; Thommen and Schumacher, 2018). Although CAR T cells are associated with several disadvantages such as their restricted efficiency, systemic immunogenicity, undesirable toxicity, and high cost as well as the extensive time that is required for production, the huge success seen in their use with hematological malignances and the continued investigations to overcome all these obstacles make CAR T cells a hugely promising therapy to treat cancers. Nevertheless, all immunotherapeutic approaches including immune checkpoint inhibitors are still at their initial steps of development and, therefore, are associated with challenges that have to be further studied and resolved, including Treg induction, toxicity, primary as well as acquired resistance, and limited efficiency.

Most of the reported clinical trials using immunotherapeutic approaches to target CSCs mainly rely on loading CSCs isolated from cancerous tissues into DCs and then transferring the DCs to the patients as a vaccine. The list of the available immunotherapy targeting CSCs can be found at http://clinicaltrials.gov, and more details can be found in the following reference (Wefers et al., 2018). As this review mainly focuses on CAR T cells in targeting CSCs, the following sections discussed CAR T cells in details.

### Genetically Engineered CAR T Cells: Production, Generations, and Signaling

**FIGURE 1:**
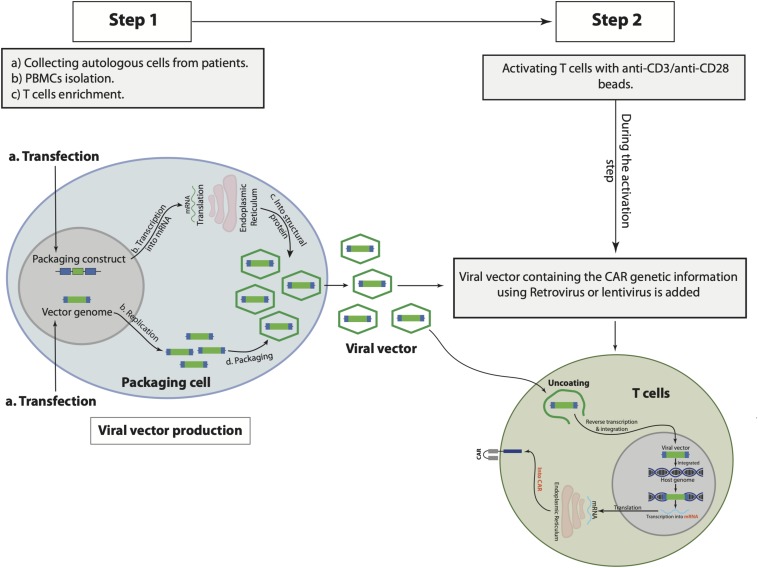
The general steps to produce and manufacture chimeric antigen receptor (CAR) T cells. Starts with collecting autologous cells from the patient, peripheral blood mononuclear cells (PBMCs), and T cells isolations from the collected autologous cells (step 1), followed by T cell activation and viral vector transfection (step 2).

CAR consists of three domains: extracellular domain, which binds to the target antigens, transmembrane domain, and intracellular signaling domain (Kuwana et al., 1987; Gross et al., 1989; Finney et al., 1998; Maher et al., 2002; Sadelain et al., 2013). Engineering CAR T cells starts with the collection of autologous cells from the patient and, subsequently, T cell enrichment and pure isolation by various methods, including gradient density to isolate peripheral blood mononuclear cells (PBMCs) and magnetic-bead-labeled antibodies to purify T cells (Powell et al., 2009; Riddell et al., 2014; Levine et al., 2017). During T cell activation *in vitro* mainly with anti-CD3 and anti-CD28 antibodies-coated beads, the viral vector using murine retroviruses or lentiviruses is added to the activated T cells (Levine et al., 2017). The viral vectors to produce CAR T cells express the genes responsible for the viral infection pathway without the genes that are associated with the virus toxicity and replication (Thomas et al., 2003). To produce viral vector, the unwanted encoding regions for virus toxicity and replication in the virus genome are deleted, while the sequences that are needed for packaging the virus capsid from the vector genome or required for the viral DNA integration are left intact in the virus genome (Thomas et al., 2003). The CAR genetic materials are then cloned into the viral genome replacing the deleted genes producing vector genome encoding CAR genetic information. A separate packaging constrict is used to aid in the replication of the modified viral genome in the packaging cells, in which the deleted genes encoding the viral replication as well as the viral capsid proteins are included in this constrict (Thomas et al., 2003). Subsequently, both the vector genome plus the packaging constrict are cotransfected into a packaging cell line and expressed as recombinant viral vector particles. The RNA of the produced recombinant viral vector is reverse transcribed into DNA, which in turn integrates the genome of the patient T cells permanently to maintain CAR expression as the cells proliferated and increased in numbers in a bioreactor (Levine et al., 2017). Subsequently, the integrated CAR DNA is then transcripted into messenger RNA (mRNA) and eventually translated into CAR expressed on the surface of the patient T cells (Figure 1). The optimal number of recombinant viral vectors to transduce and integrate the specific CAR sequence into the T cells, known as multiplicity of infection (MOI), always has to be optimized to obtain the highest expression level of CAR in T cells. It would require long-term monitoring to determine the level of safety of using viral vectors in CAR T cells, however, no reported adverse events to viral vectors have been documented so far (Cavazzana-Calvo et al., 2010; Aiuti et al., 2013; Biffi et al., 2013; McGarrity et al., 2013; Sessa et al., 2016). Notably, one of the CAR T cell therapy limitations is the persistence of the cells that might be due to the integration nature of the viral vector (Maude et al., 2014b). Moreover, patients that have received CAR T cells of viral-based vectors, namely lentiviral, might test positive for HIV. Therefore, several other approaches have been used to generate CAR T cells such as the Sleeping Beauty transposon system or mRNA transfection, however, engineering CAR T cells using viral vector, particularly lentivirus, as discussed above, is considered the most effective until now. Table 2 illustrates the advantages and disadvantages associated with each CAR T-cell-producing approach.

**TABLE 2:** The advantages and disadvantages associated with the approaches to produce chimeric antigen receptor (CAR) T cells.

Approach to generate CAR T cells	Main advantages	Main limitations
Viral lentiviral vector	• High transgene expression.	• Expensive.
	• High transduction efficiency.	• May induce oncogenesis.
	• Persistent gene transfer.	• Low inflammatory potential.
	• Integrate genetic materials stably into host genome.	• Has to be tested for safety to ensure the absence of virus replicating competent.
	• Well established system.	• Requires cells pre-activation.
		• May induce low level of mutagenesis.
Transposon	• Inexpensive.	• Low transduction efficiency.
	• Safer than viral vectors (lower genotoxicity and less immunogenetic).	• Still under development.
	• Stable genetic integration.	• Unknown potential for mutagenesis
		• Remobilization of the transposons.
mRNA transfection	• Transfect resting nonproliferating cells.	• Unstable transient expression, therefore requires several cycles of treatment (low transgene expression).
	• Do not integrate into host genome, therefore associated with very limited mutagenesis and no genotoxicity.
	• The easiest and the safest.

**FIGURE 2:**
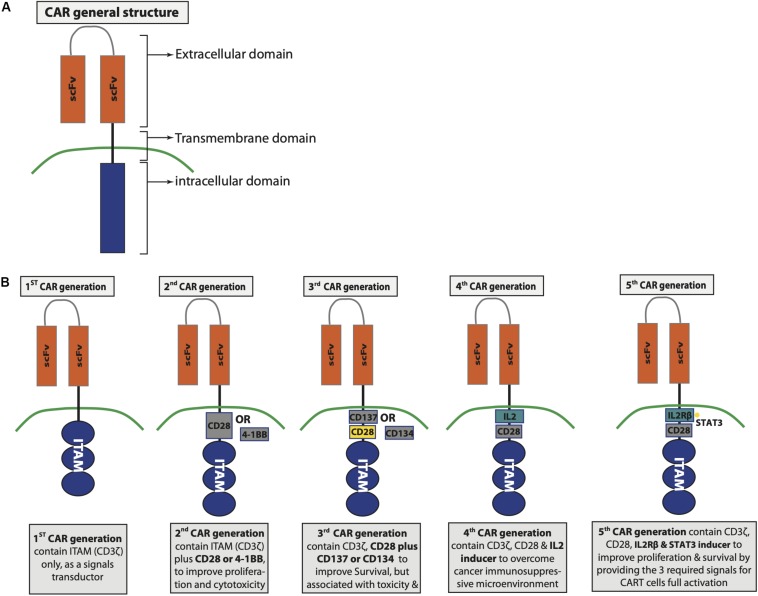
Chimeric antigen receptor (CAR) structure and generations. **(A)** General CAR structure. **(B)** Differences between the developed CAR generations.

The extracellular domain of CAR consists of a single-chain variable fragment (scFv), which is derived from the variable heavy and light regions of a tumor-specific antibody (Zhang C. et al., 2017; Ti et al., 2018). A linker that is flexible and attached via a spacer to the transmembrane domain separates the variable light and heavy chain of the scFv (Zhang C. et al., 2017). The process of CAR development witnesses several evolutions dividing the CAR into five generations, with each generation showing some genetic modifications in their intracellular domain (Figure 2). The intracellular domain of the first generation of CAR contains CD3ζ domain only (Tokarew et al., 2019), while the intracellular domain of the second generation is composed of CD3ζ plus costimulatory domain such as CD28 or 4-1BB to improve CAR T cell proliferation and cytotoxic capability (Finney et al., 1998; Hombach et al., 2001; Acuto and Michel, 2003). The third CAR generation has a similar intracellular domain to the second generation with an additional costimulatory molecule to contain two costimulatory molecules instead of one, such as CD28 plus CD137 or CD134 (Zhang C. et al., 2017). The fourth generation is also based on the second generation but replacing the additional costimulatory molecule of the third generation with protein inducer such as IL-12 (Tokarew et al., 2019). The fourth CAR generation was genetically produced to overcome the immunosuppressive microenvironment induced by tumor (Tokarew et al., 2019). IL-12 is capable of inducing IFN-γ as well as granzyme B and perforin by T cells; moreover, it has the ability to inhibit Treg proliferation (Kubin et al., 1994; Cao et al., 2008). Therefore, having IL-12 to be expressed upon CAR T cell activation increased CAR T cells’ anticancer activity. A fifth CAR generation based on the second generation is under development to include IL-2 receptor β-chain domain and binding site for STAT3 (Tokarew et al., 2019). Activating CAR T cells through the newly designed scFv provides the three signals that are required for T cell activation such as TCR signal via the CD3ζ domain, costimulatory signal through CD28 domain, and cytokine signaling via the IL-2 and STAT3 domains (Kagoya et al., 2018). CAR T cell activation via their scFv initiates cascade of signaling pathways. The most important three signaling pathways involved with CAR T cell activation includes CD3ζ, CD28, and CD137 signaling that are discussed below.

The intracellular signaling event following CAR binding to the target CSC antigen is the clustering of CAR intracellular domain, as well as the phosphorylation of the three immunoreceptor tyrosine-based activation motif (ITAM) residues of the CD3ζ and the recruitment of the subsequent downstream signaling proteins (Cantrell, 2002; Su and Vale, 2018). The phosphorylated ITAM of the CD3ζ domain interacts with the kinase, CD3ζ-associated protein kinase of 70,000 MW (ZAP70) (Hamerman and Lanier, 2006). In TCR-activated T cells, ZAP70 interaction with phosphorylated ITAM induces major configurational changes in ZAP70 that leads to their consequent interaction with lymphocyte-specific protein tyrosine kinase (Lck), which facilitate ZAP70 phosphorylation and full activation (Williams et al., 1999; Brdicka et al., 2005; Klammt et al., 2015). The fully activated ZAP70 is released from TCR/CD3 complex to the cell plasma membrane to phosphorylate its substrates such as linker for the activation of T cells (LAT) and the SH2-domain-containing leukocyte protein of 76,000 MW (SLP-76) (Katz et al., 2017). The phosphorylated LAT/SLP-76 subsequently partner up with phospholipase C-γ1 (PLCγ1) forming LAT/SLP-76 signalosome and the eventual T cell activation, proliferation, as well as differentiation (Tomlinson et al., 2000). However, the signaling pathway involved in CAR T cell activation via CD3ζ is not fully clear, but it is suggested to rely on the interaction between ZAP70 and CD3ζ ITAM (Mukhopadhyay et al., 2013; Ngoenkam et al., 2018; Ti et al., 2018).

The signals mediated via the costimulatory domain of CAR upon CAR T cells binding to antigens are mainly to improve CAR T cell functionality. CAR binding to specific antigen not only induce ITAM phosphorylation but also the phosphorylation of the tyrosine residues of the CD28, which is included in the intracellular CAR domain (Alegre et al., 2001). The phosphorylation of CD28 domain is mediated by PI3K, followed by growth factor receptor-bound protein 2 (Grb2) recruitment, protein kinase B (PKB/Akt) activation, and the eventual IL-2 production (Alegre et al., 2001; Oberschmidt et al., 2017). The third generation of CAR cells were genetically improved to include additional costimulatory domain such as CD137 to enhance the cell proliferation and survival (Pule et al., 2005; Wang et al., 2007). CD137 is expressed on activated T cells, and upon binding to its legend, the TNFR-associated-factor (TRAF) family including TRAF-1, TRAF2, and TRAF3 are recruited to the CD137 intracellular domain engaging several proteins forming CD137-signalosomes, promoting T cell survival and proliferation (Zapata et al., 2018). Although it has been reported that the functionality of CD137 included in CAR depends on TRAF-1, TRAF2, TRAF3 as well as NF-κB activation (Li G. et al., 2018), however, it is not fully clear if CD137 associated with CAR undergoes a similar response controlling molecular mechanism as CD137 of naive T cells (Zapata et al., 2018). It is extremely important to understand the controlling mechanism of CD137 signaling since it has been reported that signaling derived from CD137 domain of tonic CAR T cells leads to T cell toxicity due to the continues activation of NF-κB by TRAF2 as well as an increase in Fas killing mechanism (Gomes-Silva et al., 2017). However, the CD137 domain plays a key role in improving CAR T cell survival and efficacy, but it has to be considered that unrestricted CD137 activation may be harmful to the cells.

### CAR T Cells in Targeting CSCs and Cancer Cells

Although CAR T cells as an immunotherapy in ALL and chronic lymphoid leukemia (CLL) is promising, to date, no CAR T cell targeting CSCs have been approved. As with any treatment, CAR T cells are associated with several advantages as well as disadvantages. The most common advantage with using CAR T cells includes their ability to specifically lyse the target cells independently of MHC molecules, however, CAR T cell treatment could be associated with toxicity, CRS, and soluble tumor syndrome (Guo et al., 2018). To date, very limited number of reports, mostly in animal models, have been published on CSC targeting by CAR T cells. As mentioned above, several antigens have been identified to target CSCs by CAR T cells such as CD133, EpCAM, CD90, and much more (Guo et al., 2018). The pre-clinical and clinical trials as well as the most attractive markers for targeting by CAR T cells are discussed below in terms of relevance and features influencing CAR T cell efficiency.

Preclinical studies testing CSC-specific CAR T cell efficiency, cytolytic activities, and CAR molecule expression must be performed before utilizing these cells as a therapy. For this purpose, xenograft models have been used to evaluate CAR T cells *in vivo*, including line-derived xenograft (CDX), patient-derived xenograft (PDX) models, and models where fresh patient tumor tissues are transplanted into immunodeficient mice (Julien et al., 2012; Rosfjord et al., 2014). A study by Zhu et al. (2015), has found that CSCs isolated from GBM patients were successfully killed by anti-CD133 CAR T cells both *in vitro* and *in vivo* models of orthotopic tumor. However, CAR T cells upon their direct interaction with glioblastoma stem cells that express CD57 become functionally impaired due to the terminal effect of CD57 on T cell differentiation (Zhu et al., 2015). Moreover, Deng et al. took the lead in generating anti-EpCAM CAR cells to target prostate CSCs. In the latter study, two lines of different tumors were used: PC3 that expresses low levels of EpCAM and PC3M that express high levels of EpCAM. In their settings, PC3M cells were eliminated upon using anti-EpCAM CAR cells *in vivo* and *in vitro*. Although PC3 express low levels of EpCAM, anti-EpCAM CAR cells were able to inhibit the tumor growth of PC3 cells and to prolong the animal survival (Deng et al., 2015). Subsequent study has shown that CAR T cells targeting EpCAM on human ovarian and colorectal cancer cells are capable of killing the cancer cells *in vitro*, and the adaptive transfer of these CAR T cells prolonged the animal survival by eliminating the established ovarian xenografts (Ang et al., 2017). In agreement with these studies, a recent report has documented that the adoptive transfer of CAR T cells targeting cells expressing EpCAM significantly downmodulated the cancer growth in the xenograft model with high level of safety and no associated toxicity (Zhang et al., 2019). A generation of CAR T cells targeting EGFR were engineered by Li H. et al. (2018), which, upon testing, showed antitumor as well as expansion capabilities *in vitro* and prolonged the survival of immunodeficient mice bearing human lung cancer cells, by reducing the cancer tumor burden with no associated toxicity. In the same year, Dong et al., have also generated CAR T cells specific for EGFR but have tested their preclinical capability for hypopharyngeal squamous cell carcinoma *in vitro* only. In their setting, they have found that their generated EGFR-CAR T cells have high cytotoxic potential compared to their control cells with a lysis rate of 52.66% (Dong et al., 2018). Although most of the preclinical trials have shown a success in using CSC-specific CAR T cells by either prolonging the animal’s survival, inhibiting the tumor growth, or both, clinically, the success of CAR T cells in solid tumors was limited to feasibility with minimal efficiency due to several factors such as CSCs plasticity and heterogenicity in patients. For example, in clinical oncology, two patients of the same tumor subtype can behave differently to treatment due to their genetic differences leading to interpatient heterogeneity. However, more investigations are required to overcome all the obstacles associated with using immunotherapeutic approaches in solid cancers.

CD133 has been identified as one of the most abundant surface antigens that are highly expressed on several types of cancer CSCs including liver, brain, ovarian, lung, colorectal, and gastric (Yi et al., 2008; Baba et al., 2009; Hibi et al., 2010; Alamgeer et al., 2013; Yamashita and Wang, 2013; Zhang et al., 2014). Moreover, clinical studies have shown that CD133 expressions are extremely associated with disease resistance to treatment and poor prognosis (Zhang et al., 2010; Dragu et al., 2015). Nevertheless, the reports regarding CD133 suitability as CSC marker for certain tumors are still conflicting (Beier et al., 2007; Wang et al., 2008; Barrantes-Freer et al., 2015; Brown et al., 2017). For instance, in GBM, CD133 expression on CSC has been controversial (Bradshaw et al., 2016). It has been reported that human CD133^+^ GBM cells are capable of initiating brain tumor upon their transfer into immunodeficient mice (Singh et al., 2003, 2004). However, it was also found that CD133^–^ stem-like cells possessed similar potential of growing tumor successfully in a xenograft model (Beier et al., 2007; Shmelkov et al., 2008; Wang et al., 2008). The fact that CD133 is highly expressed in many cancers, plus it was found to be overexpressed in 50% of HCC, pancreatic, and gastric cancer patients (Ferrandina et al., 2009; Schmohl and Vallera, 2016), and highly expressed with poor prognosis, particularly in HCC (Kohga et al., 2010; Yang et al., 2010) have made CD133^+^ cells an attractive target for immunotherapy using CAR T cells. Targeting CD133-expressing CSCs with CAR T cells, regardless of the limitations stated earlier, would be of a great potential, however, few studies have investigated anti-CD133 CAR T cells in eliminating CSCs and treating cancer. A study has reported phase I trial using CD133-CAR T cells as antitumor for 23 patients of different cancers, including patients with HCC, pancreatic and colorectal cancers. The trial outcomes were reported between partial remission and stable disease with controlled toxicity (Wang et al., 2018).

Another highly expressed surface marker on many CSCs of several caner types is CD90 (Sukowati et al., 2013; Tang et al., 2013; Zhu et al., 2014; Khan and Mukhtar, 2015; Wang et al., 2015; Woo et al., 2015). CD133 and CD90 share many features including the crucial role in CSC self-renewal, CSC differentiation, and growth (Sukowati et al., 2013; Guo et al., 2018). Moreover, they regulate the oncogenesis of numerous carcinogenic diseases (Sukowati et al., 2013; Guo et al., 2018). In GBM, CD90 has been used for years as a marker for GBM CSCs (Kang and Kang, 2007; Tomuleasa et al., 2010; He et al., 2012; Nitta et al., 2015). However, CD90 expression was not found to be restricted to CSCs of GBM; it is also expressed by mesenchymal stem-cell-like pericytes, GBM-associated stromal cells, tumor-migrating cells, tumor-associated endothelial cell, neuronal cells, and by differentiated GBM cells (Clavreul et al., 2012; Ochs et al., 2013; Avril et al., 2017; Darmanis et al., 2017; Sauzay et al., 2019). Regardless of the high and consistent expression of CD90 in several cancers, CD90 expression on the CSCs of certain tumors has been controversial, particularly in renal cancer. Although CD90 is highly expressed in CSCs expressing CD105 in renal cancer, it is not detected in CSCs of patients with clear renal cell carcinoma (Bussolati et al., 2008; Galleggiante et al., 2014; Khan et al., 2016). However, high expression of CD90 in the CSCs of various cancers, including liver cancer, could be a reason to target CD90^+^ cancer cells by CAR T cells; unfortunately, no studies using anti-CD90 CAR T cell as a potential treatment for cancer have been reported.

High expression of EpCAM has been reported to play a key role in breast, head and neck squamous cell carcinoma, and colon cancers progression, as reported with CD133 and CD90; EpCAM is crucial for CSC proliferation, differentiation, and renewal (Visvader and Lindeman, 2008; van der Gun et al., 2010). Moreover, EpCAM is reported to be involved in the spread of breast as well as retinoblastoma cancers (Osta et al., 2004; Mitra et al., 2010). In HCC, several studies have shown that EpCAM is enriched in CSCs of HCC origin and that EpCAM-expressing HCC cells share more stem cell characteristics, have greater invasive, as well as tumor formation ability compared with EpCAM-negative cells (Schmelzer and Reid, 2008; Yang et al., 2008; Yamashita et al., 2008, 2009; Kimura et al., 2010; Li et al., 2016). EpCAM is also overexpressed in colorectal CSC, and it is commonly used with CD44 to identify colorectal CSCs (Dalerba et al., 2007; Liu D. et al., 2014). Several studies have reported that leucine-rich-repeat-containing G protein coupled receptor 5 (Lgr5) can be added to improve the identification panel of colorectal CSCs (Kemper et al., 2012; Jiang et al., 2016). Although EpCAM was also reported to be overexpressed in some types of cancers including breast, prostate, and pancreas, it was not detected in CSC of other cancers such as GBM (Macarthur et al., 2014). A Chinese trial has been conducted using EpCAM-CAR T cells on patients with liver cancer (Liu et al., 2017; Zhang et al., 2019). However, most of the trials are ongoing, and to date, no documented report has been published.

Several studies have reported that cancer cells that have undergone epithelial-to-mesenchymal transition possess more stem-cell-like characteristics, express an increased level of CD44 (Mani et al., 2008), and require CD44v switch to CD44s isoform (Brown et al., 2011; Zhao et al., 2016). Moreover, multiple studies have documented that CD44v expression is associated with metastasis and poor prognosis of several types of solid cancers (Mulder et al., 1994; Kaufmann et al., 1995; Li et al., 2014; Ni et al., 2014; Ozawa et al., 2014; Todaro et al., 2014). In agreement with CD44s and CD44v roles, it was found that increased levels of CD44v, in particular CD44v6, is associated with pancreatic cancer metastasis and more restricted to the late clinical stages of the disease (Rall and Rustgi, 1995; Castella et al., 1996). CD44v6 was stained positive in 50% of tissues isolated from pancreatic cancer patients, while 38% of the tissues obtained from 42 separate patients were positive for CD44v2 but not detectable in healthy tissues. Moreover, the presence of CD44v6-positive tumor cells in patients with primary cancers had given the patient shorter survival rates compared to patients with CD44v6-negative tumor tissues (Gotoda et al., 1998). CD44s was underexpressed in surgically removed specimens from patients with prostate cancers, however, the other isoforms were overexpressed. Independently, increased expression of CD44v2 was associated with improved recurrence-free rate of survival (Moura et al., 2015). To date, no clinical trial has reported CD44-CAR T cells data to treat solid tumors.

Using CD44^+^, CD24^–^, and increased ALDH activity has become the “golden standard” method to phenotype the breast CSCs (Park et al., 2010; Gong et al., 2017; Park et al., 2019). In agreement, tissues from breast cancer patients of triple-negative breast cancer (TNBC), the most aggressive form of breast cancer, showed CD44^+^, CD24^–^, and high ALDH1 phenotype compared to the nonTNBC tissues (Honeth et al., 2008; Li et al., 2013; Ma et al., 2014). Moreover, it was found that cancer cells that survive chemotherapeutic approaches in TNBC patients were of CD44^+^, CD24^–^, and high ALDH1 phenotype and showed more improved mammosphere-forming capacity (Tian et al., 2018). This similarly applies to lung cancer, where ALDH1 plus several other CSC markers including CD44 and CD133 have been identified as markers for lung CSC, but due to the heterogeneity and plasticity of lung cancer, having a specific marker for lung CSC is difficult. However, several studies have shown a strong positive association of ALDH1 with lung cancers, and inhibiting ALDH1 has led to the downregulation of stemness-related genes associated with lung cancer (Jiang et al., 2009; Leung et al., 2010; Gomez-Casal et al., 2013; Duan et al., 2014; Hardavella et al., 2016; Zakaria et al., 2017). ALDH1 has also been recognized as an CSC marker in head and neck cancer, in which an increased ALDH1 activity was associated with enhanced tumorigenesis and greater resistance to chemotherapy. Although ALDH1 has been suggested as a great marker to target CSC by CAR T cells, no study has been reported yet. However, the marker was used successfully to eliminate ALDH^bright^ cells obtained from various cancer cell lines including head and neck, breast, and pancreatic cancer lines *in vitro* with ALDH1A1-specific CD8^+^ T cells. Upon adoptive transfer of ALDH1A1-specific CD8^+^ T cells into xenograft-bearing immunodeficient mice, ALDH^bright^ cells were selectively eliminated, cancer growth and metastases were inhibited, and animals’ survival were prolonged (Visus et al., 2011). The same approach was also used by Luo et al. (2014) in which ALDH^bright^-specific CD8^+^ T cells were generated ensuing the inhibition of lung tumor cell line growth as well as prolonging the animal survival.

As discussed earlier, EGFR, in particular EGFR^VIII^, is rarely expressed in healthy tissues, characterizing this exclusive tumor-mutated receptor as an attractive therapeutic molecule. Emlet et al. (2014), have characterized GB CSCs as EGFR^VIII+^/CD133^+^ cells with self-renewal as well as cancer initiation capabilities. Moreover, they have found that EGFR^VIII+^/CD133^+^ cells can maintain EGFR^VIII+^/CD133^+^ phenotype and stem-like characteristics in tumor sphere culture, but not in standard cell culture. EGFR^VIII^ was also found to be coexpressed with undifferentiated cell markers, and upon eliminating EGFR^VIII+^/CD133^+^ cells by antibodies of bispecific property in tumor-bearing mice, the tumor generation was inhibited and the mice survival was significantly prolonged (Emlet et al., 2014). For all of the above-mentioned appealing reasons, EGFR^VIII^ was targeted by CAR T cells in patients with EGFR^VIII+^ recurrent GBM; this first clinical trial was done in 10 patients who had been on different therapeutic regimes prior to receiving EGFR^VIII^-CAR T cells. Although one patient on the trial has not shown the need for any further therapies for more than 18 months postreviving CAR T cell infusion, no noticeable tumor regression has been reported by MRI in any of the other patients. This might be due to the high heterogeneity of EGFR^VIII^ expression as well as the presence of tumor immunosuppressive microenvironment, which was worsen by postCAR T cells infusion (O’Rourke et al., 2017). Furthermore, the outcome of an additional study by Goff et al. (2019) on 18 patients with recurrent GBM who had different therapeutic interventions prior to receiving their EGFR^VIII^-specific CAR T cell infusions was not successful, a harbinger of additional barrier in using CAR T cells for treating patients with solid cancers. Moreover, Feng et al. (2017), have tested CAR T cells targeting both EGFR and CD133 to treat one patient with cholangiocarcinoma. Upon the initial infusion of EGFR-CAR T cells, the patient showed partial response of 8.5 months and extra 4.5 months upon receiving CD133-CAR T cells. However, their treatment where associated with CAR T-EGFR resistance and some degree of toxicity, suggesting that regardless of the effectiveness seen, more investigations to improve the adverse side effects are needed (Feng et al., 2017).

**FIGURE 3:**
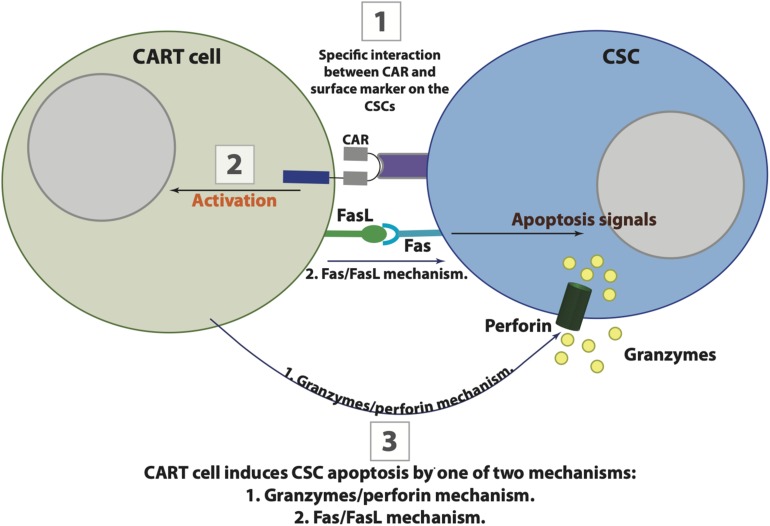
The possible interaction between chimeric antigen receptor (CAR) T cells and cancer stem cells (CSCs). CAR T cells target CSCs in three possible steps that are initiated by CAR binding to their specific antigenic target on CSC (1), followed by CART cells activation (2), and the eventual apoptosis of CSC by one of two killing mechanisms including Fas-FasL or granzymes/perforin (3).

Regardless of the initial failure seen upon using CAR T cells to treat metastatic solid tumors, several subsequent studies have confirmed the efficiency of infused CAR T cells in treating primary as well as metastatic tumors. One of the first clinical trials to examine CAR T cells was done to treat metastatic renal cell carcinoma by generating carbonic anhydrase IX (CAIX)-specific CAR T cells. Although the patients enrolled in the study have shown moderate antitumor activity as well as initial tolerance to treatment, upon several infusions, patients showed an increase in their liver enzymes, and due to the toxicity associated, the therapy was ceased (Lamers et al., 2006, 2013). Subsequently, Morgan et al. (2010), used CAR T cells to target HER2 in treating a patient suffering from metastatic colon cancer; however, the treatment was associated with fatal toxicity. Nevertheless, local delivery infusions of IL13Rα2-specific CAR T cells into a patient with recurrent GBM showed no toxic side effects and was associated with the regression of the primary as well as the metastatic spine tumors for 7.5 months. Although none of the initial primary or metastatic tumor recurred, the patient develop tumor at several new locations after a while. This was justified by some preliminary data showing that the new locations possess reduced expression of IL13Rα2 (Brown et al., 2016). The locally infused CAR T cells’ potential to prevent adenocarcinoma liver metastases (LM) was also tested by targeting carcinoembryonic antigen (CEA), a protein overexpressed in most epithelial cancers. The study included six patients who received CEA-CAR T cells with/without IL-2 supplement. Among the patients, five died from progressive disease, while one of them survived with a stable disease for 23 months posttreatment, however, all six patients have tolerated the treatment without signs of toxicity. Moreover, biopsies from some of the patients showed an increase in LM necrosis, and patients who received combined therapy documented 37% decrease in their CEA serum levels (Katz et al., 2015). Preclinical studies testing CAR T cell efficiency against metastatic cancers include a recent study showing that local infusion of CAR T cells specific for HER2 into orthotopic xenograft models has high antitumor activities against breast to brain metastases (Priceman et al., 2018). Additional preclinical study in pulmonary xenograft models has shown that vascular endothelial growth factor receptor-1 (VEGFR-1)-CAR T cells coexpressing IL-15 are able to prevent pulmonary metastasis (Wang W. et al., 2013). In a lung cancer model, CAR T-cell-targeting tissue factor (TF), found to be overexpressed in squamous cell carcinoma and adenocarcinoma of nonsmall cell lung cancer as well as melanoma, suppressed the cancer in the xenograft and prevented the metastasis of TF-expressing tumor cells without associated toxicity (Zhang Q. et al., 2017). Recently, Seitz et al. (2020) have generated CAR T cells targeting disialoganglioside GD2, a breast CSC marker, and reported that their generated CAR T cells are capable of preventing the tumor progression as well as the formation of lung metastasis in an orthotopic xenograft model of TNBC. Few studies have been published reporting the efficiency of CAR T cells in preventing metastatic prostate cancer (mPCa) mainly by targeting prostate-specific membrane antigen (PSMA), which is expressed in prostate cancer cells. In a preclinical setting, Zuccolotto et al. (2014), have targeted human PSMA by CAR T cells in prostate tumor-bearing mice, reporting the complete elimination of metastatic cancer cells in majority of the animals. Clinically, Slovin et al. (2013), conducted a phase I trial using CAR T cells specific for PSMA in patients with castrate metastatic prostate cancer. Some patients were stable after receiving the treatment, while others had progressed disease, and the degree of toxicity were dose dependent. Despite all the reported studies and trials, the capability of CAR T cells to prevent metastatic spread still requires more investigations in order to reach applicable clinical conclusions. Moreover, although CAR T cells are a very appealing therapy especially with the incredible success seen in some hematological malignancies, collectively, these data suggest that solid tumor targeting by CAR T cells has a poor efficiency for several reasons and many challenges, which are discussed below. However, there is a great interest in improving CAR T cell efficiency to overcome all the associated issues with their application. Figure 3 illustrates the possible killing steps by CAR T cells, and Table 3 summarizes examples of the published clinical trials of CAR T cells in some of the solid tumors.

**TABLE 3:** Examples of the published clinical trials of chimeric antigen receptor (CAR) T cells in some of the solid tumors.

Tumor type	CSC markers	CAR T cells clinically	Results obtained clinically	References
Brain	CD133 EGFR^VIII^	EGFR^VIII^-CAR T cell	Showed success in one patient, while the others have no noticeable tumor regression.	O’Rourke et al., 2017
		EGFR^VIII^-CAR T cell	Not successful outcomes	Goff et al., 2019
		IL-13Rα2-CAR T cells	Regression of primary and metastatic spine cancer, with no toxicity, but recurrence at several new locations.	Brown et al., 2016
Prostate	EpCAM CD44 ALDH	PSMA-CAR T cells	Mixed outcomes between stability and progressed disease (toxicity was dose dependent)	Slovin et al., 2013
Colon	EpCAM, CD44, Lgr5 CD133 ALDH HER2	HER2-CAR T cell	Fetal toxicity	Morgan et al., 2010
Liver	CD133 EpCAM EGFR CD44 CD90	CD133-CAR T cells (HCC, pancreatic, and colorectal cancers)	Outcomes between partial remission and stable disease with controlled toxicity.	Wang et al., 2018
		EGFR-CAR T cells plus CD133- CAR T cells	EGFR-CAR T cells infusion showed partial response of 8.5 months and extra 4.5 months upon receiving CD133-CAR T cells, with some degree of toxicity.	Feng et al., 2017
		CEA-CAR T cells ± IL-2 supplement	One patient survived and the rest died, however, no toxicity reported.	Katz et al., 2015

### Barrier in Using CAR T Cells

CAR T cells have revolutionized the world of fighting cancers by immunotherapeutic approaches. Since the reported success of anti-CD19 CAR T cell in treating ALL and CLL and approval of the first anti-CD19 CAR T cells therapy to treat B cell ALL and diffuse LBCL by the Food and Drug Administration (FDA), the number of clinical trials targeting several antigens other than CD19 using CAR T cells has dramatically increased (Kochenderfer et al., 2010a; Kalos et al., 2011; Porter et al., 2011; Mullard, 2017; Tang et al., 2018; Shah and Fry, 2019). However, about 30–50% of patients who received anti-CD19 CAR T cells have relapsed 1 year from their remission, while 10–20% of the patients did not reach the remission phase following anti-CD19 CAR T cell treatment (Lee et al., 2015; Gardner et al., 2017; Maude et al., 2018; Park J.H. et al., 2018). Patients’ relapse following treatment with CAR T cells was not exclusive to anti-CD19 CAR T cells, as other approaches using CAR T cells, for example, to target CD22 were also associated with relapse (Fry et al., 2018). This suggest that relapse and recurrence will be a common issue associated with CAR T cell therapy, especially if they were not used to target CSCs.

As mentioned, CAR T cells’ potential in treating cancer is very promising, however, the toxicity associated with the treatment is one of the major obstacles. CAR T cell toxicity has been classified into five categories, on-target/on-tumor, on-target/off-tumor, off-target, neurotoxicity, and other toxicities (Sun et al., 2018). On-target/on-tumor is toxicity associated with T cells’ release of excessive cytokines as well as the resulted necrotic tumor cell, leading to what is known as CRS and tumor lysis syndrome (TLS), respectively. However, it has been reported that this type of risk can be minimized based on the disease burden and the appropriate monitoring as well as the suitable splitting of the doses. Since those risks are rapid immune responses of massive cytokine release, administrating a dose of corticosteroids as well as antagonist mAb can be effective (Brentjens et al., 2013; Teachey et al., 2013; Davila et al., 2014; Maude et al., 2014a; Bonifant et al., 2016; Sun et al., 2018). The most noticeable CAR T-cell-associated toxicity is due to the presence of the target CAR T cell antigen on both the tumor as well as the healthy tissues, a phenomenon known as “on-target/off-tumor” (Sun et al., 2018). This shared expression is enormously damaging because CAR T cells can target healthy tissues expressing even the lowest levels of the target antigen (Sun et al., 2018). This was seen in an early study performed at Erasmus University, where they have observed that infusing carbonic anhydrase IX-CAR T cells into patients with renal cell carcinoma resulted in cholestasis due to the physiological expression of the target antigen on the epithelial cells of the bile duct (Lamers, 2009; Lamers et al., 2013). These results were not limited to the latter study (Hombach et al., 2010); therefore, selecting target antigen for CAR T cells with the knowledge of its background expression is the most crucial to have better application as well as to decide on the threshold causing toxicity and to determine the possible severity in human (Sun et al., 2018). Recently, a novel universal CAR (uniCAR) system is developed to reduce the risk associated with on-target and to control CAR T cell reactivity, allowing CAR T cell to switch on and off in controlled approach. UniCAR system signaling and antigen-binding characteristics are separated into two independent components. T-cell-expressing uniCAR specifically recognizes human nuclear protein and consists of 10 amino acids; therefore, uniCAR cells are inactive upon infusion due to the lack of their target. UniCAR cells become activated via a separated system that bridge the uniCAR cell binding domain with its nuclear antigen motif fused to tumor-antigen-specific scFV (Cartellieri et al., 2015). Unfortunately, the use of immunodeficient model is insufficient and associated with several drawbacks that limit the assessment of toxicity such as on-target/on-tumor and on-target/off-tumor (Kochenderfer et al., 2010b). One of the challenges associated with immunodeficient model is that human-specific CAR T cells can lead to graft-versus-host disease in mice due to recognizing the mouse xeo-antigens limiting the utilization of this model in evaluating therapies targeting slow-developing cancers without understanding the practical therapeutic window for the model (Alcantar-Orozco et al., 2013). An additional obstacle associated with this kind of animal model is that the mice do not represent the clinical situation due to their limited endogenous lymphocytes. Although cancer patients usually undergo lymphocyte depletion regimens, their lymphocyte recovery occurs, developing the various populations of T cells including Tregs that downregulate the antitumor effect accompanied with the transferred CAR T cells, a situation that is not replicated in the mice model (North, 1982; Gattinoni et al., 2005). However, this model has been useful in confirming that CAR T cells are able to target tumors; the obstacles associated with solid cancer microenvironment might be undervalued (Sharpe, 2018). Therefore, animal equivalent products as well as syngeneic tumor models might be more useful in testing CAR T cells’ safety and efficacy (Kochenderfer et al., 2010b; Davila et al., 2013). CAR T cells can go out of their way attacking antigens nonspecifically, off-target toxicity; fortunately, this issue of cross-reactivity has not yet been reported upon using CAR T cells. However, it should be kept in mind while developing CAR T cells targeting certain antigens (Bonifant et al., 2016). Of the most serious toxic effects associated with CAR T cell treatment is neurotoxicity, which has been reported for no certain well-defined causative pathophysiology in patients infused with CD19-specific CAR T cells (Sun et al., 2018). Several other CAR T-cell-associated toxicities have been reported, including immunosuppression, immunogenicity, and genotoxicity. However, for more details on toxicity associated with CAR T cell immunotherapy and the possible strategies to overcome it, readers are referred to reference Sun et al. (2018).

Unlike solid tumors, CAR T cells’ systematic administration for hematological malignancies was a success because the target was easily reached by CAR T cells. One of the barriers that CAR T cells have to overcome in solid cancers is reaching their target in the tumor site. However, improving CAR T cells’ strength for systemic administration is associated with some safety concerns, as documented upon using HER2-specific CAR T cells for therapy. HER2-specific CAR T cells were generated with high-affinity form of scFv that was able to recognize even normal lung cells expressing low levels of HER2 leading to fatal pulmonary toxicity and CRS (Morgan et al., 2010). One of the possible solutions is the local administration of CAR T cells into the targeted tumor bed. For instance, the administration of IL13Ra2-specific CAR T cells intraventricularly shows intracranial and spinal tumor regression in recurrent GBM patients (Brown et al., 2016). Moreover, mRNA-transduced anti-c-Met CAR T cells were examined through intratumoral administration in a clinical trial on patients with metastatic breast cancer, and the treatment was reported to be feasible and was also associated with extensive tumor necrosis at the site of injection as well as inflammation (Tchou et al., 2017). This study was subsequently confirmed, where intraventricular administration of HER2-specific CAR T cells was reported by Priceman et al. (2018) to have more antitumor response in orthotopic xenograft models of brain metastatic breast cancer when compared to intravenous infusions. Another proposed approach is the use of what is called masked CAR (mCAR) T cells, which only get activated and unmasked upon exposure to protease, which is mostly found in the TME, not in healthy tissues. The concept of mCAR T cells was tested through generating mCAR T cells targeting EGFR that were activated against EGFR-expressing cells upon exposure to tumor protease (Han et al., 2017). CAR T cells’ inability to reach their target site is mainly due to their failure to track a chemotactic gradient due to chemokine-receptor mismatch; moreover, CAR T cell entry to the tumor site can get blocked by some physical barriers including cancer-associated fibroblast and abnormal vasculature (Hanahan and Coussens, 2012). Additionally, solid tumor usually causes damage to the blood vessels, known as high endothelial venules, which are considered as important entry points for lymphocytes (Ager, 2017). Since chemokines could play a crucial role in CAR T cells’ homing to the tumor site, “armored” mesothelin CAR T cells were generated expressing constitutive IL-7 and CCL19. These generated CAR T cells were found to completely increase tumor regression and to prolong the survival of solid tumor-bearing mice (Adachi et al., 2018). Data in this area are still being collected, with very promising results to improve and to overcome the advised side effects that are usually associated with CAR T cell systemic administration as well as toxicity.

CAR T cells as a monotherapy to treat solid tumors was associated with limited efficiency in most of the clinical trials. Therefore, one of the suggested strategies to increase the efficiency of CAR T cell therapy is to combine it with other therapeutic regimes such as chemotherapy and radiotherapy. Several studies have reported that combining CAR T cells with chemotherapy can reduce the disease-associated side effects, improve the recognition of the tumor antigens, and enhance CAR T cell efficiency and persistence (Proietti et al., 1998; Alizadeh et al., 2014; Muranski et al., 2006). This enhanced efficiency was also seen upon combining CAR T cell therapy with radiotherapy. Weiss et al. (2018) have found that combining CAR T cells with radiotherapy enhance T cell infiltration and transport, produce synergistic activity, enhance the presentation of tumor antigen, and increase CAR T cell durability. Multiple reasons might be behind the enhanced efficiencies and persistence of CAR T cells upon combining it with chemotherapy and radiotherapy, including the ability of those therapies to modify TME and to remove immunoregulatory cells facilitating CAR T cells role. Combining CAR T cell therapy was also suggested to be beneficial with checkpoint inhibitor therapy especially for patients who, postreceiving CAR T cell therapy, might experience antigen escape and subsequent CAR T cell failure and recurrent malignancies. However, this was only reported so far to be effective in mice (John et al., 2013). The reported studies on the direct effect of cancer treatment on T cells’ cytotoxic capabilities in targeting CSCs are lacking, unlike NK cells, where Luna et al. (2019), have found that bortezomib, a clinically used proteasome inhibitor to treat multiple myeloma as well as mantle cell lymphoma patients, can enhance the targeting of CSCs by NK cells through upregulating NK cells ligands, MICA and MICB expression, as well as MHC class I on the surface of ALDH^+^ CSCs. These data support the importance of using combined therapy upon transferring CAR cells, with emphasis on the need to study the exact and direct influence of other therapies that would be combined, on CAR T cell capacity in targeting CSCs. Furthermore, most of CAR T cells’ clinical trials to target CSCs have been done on patients who have failed to respond to their therapeutic regimes and are with poor physical conditions, which can be the reason behind the failure of CAR T cells as monotherapy. More importantly, it is impossible for CAR T cells as a monotherapy to eradicate heavy burden solid tumors; therefore, using CAR T cell combined with other therapies would improve the value of CAR T cell therapy, particularly if the patients were selected at early stages of the disease to increase the chance of the removal of both CSCs and nonCSCs at once.

Several other reasons have been cited as obstacles to effective CAR T cell treatment; most commonly is due to alteration or loss of the target antigen (Gardner et al., 2016; Jacoby et al., 2016; Fry et al., 2018), inconsistency of CAR T cells, as well as unsuccessful manufacturing (Mueller et al., 2017; Stroncek et al., 2017; Ceppi et al., 2018). Apart from the success reported with CAR T cells in B cell leukemia and lymphoma, no other diseases have documented this achievement with CAR T cells regardless of their wide use as a targeting therapy. Therefore, understanding the limitations of these cells as a therapy and solving the issues associated with their application is crucial to benefit fully from such powerful approach.

## Conclusion

The fact that CAR T cells can target any molecule in a cell, independently of MHCs, made CAR T cells targeting CSCs very attractive and a powerful tool, particularly for hematological malignances. Unfortunately, most of the clinical trials using CAR T cell to target CSCs in solid tumors have been disappointing due to several challenging barriers, including toxicity, CRS, soluble tumor syndrome, alteration or loss of the target antigen, as well as unsuccessful manufacturing. Therefore, many groups have tested several strategies to overcome these issues, for example, infusing CAR T cell locally instead of systemically to improve safety and minimize CAR T cell on-target/off-tumor adverse side effects. Moreover, several steps have been taken to upgrade CAR T cells including the generation of uniCAR T cells. However, using CAR T cells to target CSCs will always be associated with obstacles, unless a stable and unique target is identified to differentiate CSCs from the rest of the tumor as well as healthy cells. CAR T cells’ future in targeting CSCs is still under investigation, and many studies are needed to both identify the uniquely expressed targets as well as to improve CAR T cell production and administration regimes.

## Author Contributions

RA collected data from published studies and wrote the manuscript.

## Conflict of Interest

The author declares that the research was conducted in the absence of any commercial or financial relationships that could be construed as a potential conflict of interest.
